# Female Genital Mutilation Consequences and Healthcare Received among Migrant Women: A Phenomenological Qualitative Study

**DOI:** 10.3390/ijerph18137195

**Published:** 2021-07-05

**Authors:** Alba González-Timoneda, Marta González-Timoneda, Antonio Cano Sánchez, Vicente Ruiz Ros

**Affiliations:** 1 Faculty of Nursing and Chiropody, University of Valencia, 46010 Valencia, Spain; vicente.ruiz@uv.es; 2 Department of Obstetrics and Gynecology, University Clinic Hospital, 46010 Valencia, Spain; mgonzaleztimoneda@gmail.com; 3 Faculty of Medicine and Dentistry, University of Valencia, 46010 Valencia, Spain; antonio.cano@uv.es

**Keywords:** female genital mutilation, female circumcision, health consequences, women’s health, nursing, midwifery, migration, health equity, qualitative research

## Abstract

European healthcare systems are increasingly being challenged to respond to female genital mutilation (FGM). This study explores the FGM experiences of migrant women coming from FGM-practicing countries residing in a European host country. A qualitative phenomenological study was carried out and 23 participants were included. Data were collected through 18 face-to-face open-ended interviews and a focus group and were analysed using Giorgi’s four-step phenomenological approach. Three main themes were derived: “FGM consequences”, “healthcare received” and “tackling FGM”. Participants highlighted obstetric, gynaecological and genitourinary consequences such as haemorrhages, perineal tears, caesarean delivery, risk of infection, dysmenorrhea, urinary tract infections and dysuria; consequences for sexuality, mainly, dyspareunia, loss of sexual interest and decreased quality of sexual intercourse; and psychological consequences such as loss of self-esteem, feelings of humiliation and fear of social and familial rejection. Women perceived a profound lack of knowledge about FGM from health providers and a lack of sensitive and empathetic care. Some women perceived threatening and disproportionate attitudes and reported negative experiences. Participants highlighted the importance of educating, raising awareness and improving prevention and detection strategies. The findings disclose the need to improve training and institutional plans to address structural and attitudinal barriers to health equity across migrant families in their host countries.

## 1. Introduction

Violence against women is one of the most pervasive gender-based inequalities that creates inequalities in many areas of the life of women and girls. These disadvantages pose serious threats to their welfare and the fulfilment of their rights [1]. The practice of female genital mutilation (FGM) is a harmful traditional practice that involves partial or total resection of the female external genitalia or other injury to the female genitalia for cultural or other non-medical reasons [2]. The typology of FGM is shown in Table 1 [2]. FGM is recognised internationally as a violation of the human rights of girls and women and as an extreme form of gender discrimination that should be eliminated to achieve gender equality and women’s empowerment. That is why the United Nations strives for its full eradication by 2030, following the spirit of Sustainable Development Goal 5 [3].

According to the latest United Nations Children’s Foundation (UNICEF) data, the exact number of girls and women affected by FGM globally remains unknown. However, at least 200 million of women and girls have undergone FGM and it is estimated that 68 million girls will undergo FGM before 2030 if action against this practice is not intensified [4,5].

FGM is mainly concentrated in 130 countries in the western, eastern and north-eastern regions of Africa, along with the Middle East and is practised in some countries of Asia and Latin America with wide variations in prevalence [6]. Nevertheless, not all ethnic groups living in these countries practice FGM, nor do all the ethnic groups that practice it follow the same procedure. The kind of FGM carried out varies mainly according to ethnicity [7]. FGM is carried out during infancy with most girls cut before the age of 15. In others, it takes place at the time of marriage, during a woman’s first pregnancy or after the birth of her first child [5,7].

Thus, although the practice is originally characteristic of certain areas, this local phenomenon has been globalised and spread around the world through the different migratory movements. Therefore, FGM and its harmful consequences are affecting a growing number of women and girls among migrant communities in Europe, North America and Australia [5]. According to the 2011 census, around 600,000 women and young females have experienced FGM in Europe and it is considered that 190,000 young girls are at risk of FGM in 17 European countries alone [8]. In the United Kingdom (UK), it was estimated that 137,000 women and girls were living with FGM in England and Wales in 2015 [9]. In Spain, it is estimated that almost 70,000 women and girls come from countries affected by FGM [10]. The Valencian Community is the fourth Spanish autonomous community with the largest population from countries affected by FGM; mainly from Nigeria, Senegal, Guinea, Mali and Cameroon [10].

It is expected that the number of women with FGM in Europe will rise at quite a fast rate and future flows are expected to be strongly geographically selective, involving mainly France, Italy, Spain, the UK and Sweden [11]. This fact has implied that healthcare systems in European countries are increasingly being challenged to respond to the care of affected communities.

The practice is painful and traumatic and its performance is often unhygienic or carried out by non-expert practitioners who have little knowledge of the anatomy of the genitourinary system of women and lack the ability to respond to adverse events. FGM has consequences that undermine the health and well-being of girls and women, revealing a remarkable situation on the global women’s public health agenda [12,13]. The effects of FGM depend on several factors, including the type performed, the expertise of the practitioner, the hygiene conditions under which it is performed, the amount of resistance and the general health condition of the girl/woman undergoing the procedure [12].

Immediate complications include severe pain, shock, haemorrhage, tetanus or infection, urine retention, ulceration of the genital region and injury to adjacent tissue, wound infection, urinary infection, fever and septicaemia. Haemorrhage and infection can be severe enough to cause death. Long-term consequences include complications during childbirth, anaemia, the formation of cysts and abscesses, keloid scar formation, damage to the urethra resulting in urinary incontinence, dyspareunia (painful sexual intercourse), sexual dysfunction, hypersensitivity of the genital area and increased risk of HIV transmission, as well as psychological effects [12].

In addition to the physical and psychological impact of FGM, the associated complications are usually not the primary problems for women who arrive as refugees or migrants. They are very likely to come from conflict zones where they experience poverty, malnutrition, health problems, lack of educational opportunities and restricted access to health care services [14]. This fact discovers a variety of cultural realities for health professionals that have led to face new care challenges. Furthermore, migration can create or increase vulnerability due to multiple factors such as low socioeconomic status, language, cultural barriers, or lack of knowledge about healthcare rights and accessibility. This vulnerability can be notable during the period surrounding motherhood, when these women normally tend to attend for the first time the healthcare system. Almeida et al. reported, in migrant women, lower levels of access to health care and poorer birth outcomes than non-immigrants or English-speaking immigrants [15].

Evans et al. suggests that care and communication around FGM can pose significant challenges for women and other healthcare providers [16]. Several studies have explored the knowledge and attitudes of healthcare professionals regarding the practice of FGM in western countries evidencing a profound lack of knowledge about the practice of FGM [17,18]. Evidence also indicates that women affected by FGM do not receive appropriate healthcare due to cultural barriers and lack of knowledge and capability to provide competent transcultural care by healthcare professionals [17,18].

Nonetheless, limited research has examined how women and men experienced the practice of FGM and its consequences, as well as the healthcare received within the Spanish national public health system [19,20,21,22]. Furthermore, there have been no studies to date that examine the experience of women affected by FGM when receiving healthcare in the region of Valencia, although evidence indicates that healthcare providers are not knowledgeable about FGM despite being a problem present in the Valencian community [23,24].

To address this need and considering that FGM may act as an element of discrimination and a condition for health inequalities in contexts where the practice of FGM is unknown and ignored, the aim of this study was to gain in-depth experiential knowledge about the experiences regarding FGM of migrant women coming from FGM-practicing countries residing in their European host country.

## 2. Materials and Methods

### 2.1. Design

A qualitative study design with a phenomenological approach was chosen. Phenomenology was selected on the basis of its relevance to research individual lived experiences, meaning-making and interpretation of one’s experience or perception. This method, developed by Husserlian philosophy [25], aims to explore the same phenomenon through rich descriptions of individuals revealing common features of the lived experience. For Husserl, to understand a phenomenon, whatever the purpose, it is not possible to ignore the experience of the person who lives the phenomenon [25].

### 2.2. Participants

A total of 23 participants were included in the study. For the selection of the participants a purposeful sampling strategy was used, which implies identifying and selecting individuals or groups of individuals that are especially knowledgeable about or experienced with a phenomenon of interest [26]. The sampling was based on a profile that comprised a set of selection criteria: women and men 18 years of age and older coming from countries where FGM is practiced, who had undergone FGM or had been in close contact with the practice, who were able to provide informed consent, who spoke English or Spanish or had translation assistance during the interview. Of the 23 participants recruited, 20 women had undergone FGM. We also included the two men and a woman that had been in close contact with the phenomenon studied since their wife and female relatives had FGM performed and they had lived closely the FGM consequences.

Contact with the target population was made through key informants. Key informants were local community leaders who were located via community health agents, local associations of migrants and social organizations and services. Key informants were asked if they wanted to participate in the study if they met the inclusion criteria. Key informants also served as gatekeepers since they were trusted sources that controlled access to the target population. Using the snowball sampling technique, they contacted potential participants and asked them for the first time if they would agree to the study. This technique was chosen because its suitability provides forms of contact with populations or groups characterised as difficult to access or, meant in the literature, as hard-to-reach populations [26,27] or hidden populations [28,29]. Participants who agreed to be contacted by the researchers were approached by telephone and face-to-face. In addition to being an intentional and reasoned sampling, it was cumulative, flexible and reflective as it was expanded throughout the research process until data saturation was reached.

### 2.3. Data Collection

All eligible participants who were approached agreed to be part of the study. Data were collected through 18 face-to-face open-ended semi-structured interviews and a focus group. The study was carried out in two stages: the first stage was carried out in London (United Kingdom) in January of 2017 and the second stage was carried out in Valencia (Spain) from February to May 2017. The focus group was conducted in April 2017 in Valencia. The study was carried out in two stages based on the criteria of convenience-relevance and the sufficiency of the sample, which were also considered to facilitate the research process and the scope of the saturation principle.

First, a few questions exploring the sociodemographic characteristics of the sample were undertaken. The following general questions included women’s experiences on FGM, its consequences and the healthcare received. The participants chose the times and locations for the interviews: participants’ home, workplace or a cafe or a garden close to their homes. Two researchers were involved in carrying out the interviews. The researcher’s credentials, occupation and training were informed to participants prior to data collection. All narratives were, after obtaining consent, recorded in audio format. Field notes were written after each interview to detail observations that could not be captured via the audio recording. The duration of interviews sessions ranged between 11 and 58 min.

The focus group was conducted to stimulate the interaction between the participants and to explore the discourse in certain social context, capturing the social experience and the different opinions and contractions following Berenguera approach [30]. The focus group included five Nigerian women coming from vulnerable social groups such as prostitution and trafficking who agreed to participate. They were contacted through a local women’s support association. The focus group was open, non-directive and flexible and offered the participants the freedom to answer or not the questions posed to them. For the implementation of the focus group, a second person was required to act as moderator.

### 2.4. Ethical Considerations

All participants received oral and written information about the purpose of the study, voluntary participation, guaranteed confidentiality and the right to discontinue at any time without any adverse effects. All participants signed a written informed consent prior to each interview. All interviews were coded prior to their transcription to guarantee the confidentiality of the participants. Approval was obtained from the Ethics Committee in Human Research of the University of Valencia, Spain.

### 2.5. Data Analysis

Data analysis was conducted according to the four-step phenomenological approach of Giorgi [31] and took place concurrently with data collection. The first step was data immersion; interviews transcripts and field notes were thoroughly read and digital recordings were also carefully listened to obtain a sense of the whole described by the participants. All interviews were transcribed verbatim by the lead researcher protecting all the participants identities using code numbers. The second step involved dividing narrative data into concepts which required the extraction of individual meaning units or conceptualisations. This was possible by re-reading the transcripts again breaking down the whole through analysis into common elements. Together two authors independently analysed the narratives and interviews both as a whole and for meaning. To facilitate the management and grouping of qualitative data, the Atlas.ti v.8. qualitative data analysis software was used [32]. The third step consisted in organising, analysing and transforming the language of the participants into a conceptual perspective of the experience, relative to the phenomenon of interest. This step saw the emergence of themes and the authors worked collaboratively to discuss the emerging themes and resolve any differences. In the final step, themes and sub-themes were combined into a final general description that reflected the lived experience of participants. All researchers agreed on the final thematic structure.

### 2.6. Rigour

To assess the rigour of the research process developed in the framework of qualitative research, we have based ourselves on the general criteria described by Guba and Lincoln: credibility, transferability, dependency and confirmability [33]. Specific strategies to attain trustworthiness were used as recommended by Guba and Lincoln [33]. Firstly, to obtain credibility, data are presented as verbatim quotes and explained by the authors’ interpretation to illustrate the richness of the data. In addition, the data, researchers and methods were triangulated. The member check strategy was also performed with one participant who agreed to reviewthe returned transcript. Secondly, to facilitate transferability to other settings, detailed descriptions of the context, the sample, the participants’ perspective and the phenomenon itself have been produced in order to allow readers to make informed judgements about similarity between contexts. Thirdly, to check dependability, data collection tools and analysis strategy and findings are thoroughly described. Finally, the researchers used reflexivity about their own position on the topic to ensure the confirmability of the data.

This study has been reported in line with the Consolidated Criteria for Reporting Qualitative Research (COREQ) guidelines to enhance the quality and transparency of the study [34].

## 3. Results

From the 23 experiences collected, 20 women had undergone FGM, of whom 14 had been mothers. The mean age of the men interviewed was 50 years, while that of the women was 31.8 years. All the participants came from countries affected by FGM: Mali, Somalia, Nigeria, Burkina Faso, Senegal and Cameroon, except for one participant who came from Equatorial Guinea and was included in the study since she had close contact and knowledge about FGM. The detailed sociodemographic characteristics and the FGM status of the study participants are shown in Table 2 and Table 3.

Three main themes were derived from narrative data: (a) FGM consequences, (b) healthcare received and (c) tackling FGM. From these, several sub-themes emerged, which are described below (Table 4). Representative quotations from the participants are used to verify and validate the findings.

### 3.1. Consequences of FGM

In this category, the following subthemes were derived: “obstetric consequences”, “genitourinary consequences”, “pain”, “sexual complications”, “psychological and social consequences”, “men’s consequences” and “lack of insight”. In general terms, both short and long-term consequences were described. The severity of complications depends on the type of FGM that has been performed, types II and III being the most intrusive: “The bigger the grade is, the more complications are” (I1LW). Other factors, such as the girls’ prior health or the conditions in which the practice is carried out, also influence the consequences of the procedure.

#### 3.1.1. Obstetric Consequences

Regarding obstetric consequences, the most frequently mentioned were postpartum haemorrhage, perineal tears, pain, caesarean delivery and the risk of infection. Infertility, preterm birth, shoulder dystocia and even death were also mentioned (Table 5). Serious complications such as the death of girls and women due to postpartum haemorrhages were discussed. Of the fourteen women who had undergone FGM and had been mothers, 46% ended the pregnancy by an elective caesarean section or by an urgent caesarean section due to failed induction of labour.

#### 3.1.2. Genitourinary Complications

Multiple allusions were also made to bleeding secondary to FGM itself, also associated with the significant risk of traumatic wound infection.

“In my country (Mali) they usually do FGM to babies of a week or days of life. There are not so many means to do it. Likewise, they cut 20 girls using the same knife, so there are many infections. There are also girls who die from hemorrhages…”

(I14VW)

“There are many complications (…) first, there are plenty hygienic infections”

(I8VW)

“Bleeding... Her family was healing her, at home! Not in a hospital”

(I9VW)

In addition, to wound infections caused by FGM, women also refer us to other genitourinary complications such as repetitive urinary tract infections: “Yes, many (urine infections), as a child and now” (I12VW)

#### 3.1.3. Pain

Moreover, the pain is a symptom that appears associated with most complications’ secondary to FGM. We have found references to it both when urinating (dysuria), having sex (dyspareunia), related to menstruation (dysmenorrhea) or related to birth or gynaecological examination (Table 6).

#### 3.1.4. Sexual Complications

Regarding the consequences on sexuality, dyspareunia, the decrease or absence of erotic desire, the decrease in the quality of sexual relations and anorgasmia were mainly mentioned: “Then, when you get married it is very difficult to feel pleasure” (I1LW), “She is suffering because she does not have the desire to have sex” (I9VW).

#### 3.1.5. Psychological and Social Consequences

Regarding psychological consequences, the participants did not explicitly describe suffering from anxiety and depressive disorders, post-traumatic stress, or reminiscences of the moment of the cut. However, we can find verbatims of clear components of psychosocial affectation such as loss of self-esteem, feelings of humiliation and fear of social rejection and dishonour of the family.

“I had to please my parents, I had to please everyone. I was given plenty of gifts, gold, jewellery, which is hardly praised in our culture”

(I1LW)

“Because a girl who is not mutilated, people call them ‘bilákoro’, it’s like… if you are dirty, you are not welcomed…”

(I15VM)

“It is a party, the day of FGM comes and then the whole family gets prepared. It is said that the girl’s crying defines the courage of the family”

(I15VM)

#### 3.1.6. Lack of Insight

Finally, we have also found different testimonies from women affected by FGM who report not having suffered any associated complications. Two participants expressed: “There are no complications after female circumcision” (IG); “It didn’t affect my sex life and it didn’t affect my pregnancy. I would have loved to have my clitoris hanging, but it’s not there which is fine, no problem! Sex life it’s OK! I cannot complain! (Laughs…)” (I1LW)

Many women do not perceive the consequences and complications as a cause of FGM, since they understand that they are “common and normal in women”. Therefore, they do not perceive the need to seek support, help or assistance in the face of a problem, since they consider it as something intrinsic to the nature of women.

“If they have severe FGM types, they have lots of problems such as infection, difficulty with bleeding like periods problems. However, they do not seek much help because they think that is normal for every woman, the more they are educated the more help is seek. Yes, but I do not think they will be coming in numbers. Only the ones who knows better”

(I1LW)

There is also a belief in some communities that if something bad happens, the existence of a “higher being” is what determines the final result as reported by one participant:”It is something normal in life. For example, when girls die during the FGM procedure, people think that God has made the decision and it happens because it must happen. It is the same for women who die giving birth. They do not associate it with mutilation” (I14VW)

#### 3.1.7. Men’s Consequences

Some of these consequences also affect men. For I2LW interviewee, men do not suffer any consequence: “I don’t know the men, I don’t think they have problems” (I2LW). However, other participants suggest the opposite: coping with the fear of losing virginity on their wedding night, frustration, or decreased quality of sexual relationships.

“A lot of pressure comes also from men, but I think in my culture lots has changed, and men are stepping back, and they don’t want to go through that trouble (…), when a woman who is FGM type III comes to them, and they have to open their vagina with their own genitalia”

(I1LW)

“There are men who do not want to sleep with the woman that day, but there are others who force her wives because they have to do so (…)”

(I14VW)

“Women do not have pleasure when they have sex and this generates many frustrations, for men too, so they do not feel satisfied in bed and then the problem begins. They go with other women…”

(I14VW)

### 3.2. Healthcare Received

From this theme, four subthemes were derived: “unacquainted professionals”, “lack of detection and information”, “stigmatizing and over-inquisitive attitudes” and “offering reversal or deinfibulation”.

#### 3.2.1. Unacquainted Professionals

The lack of knowledge about FGM of different professionals perceived by women emerged, for example, when they underwent gynaecological examination or received childbirth care.

“I knew that the way they looked at me meant that they had no knowledge of what had happened to me (…) When we talk between us, the ignorance of professionals comes up”

(I14VW)

The interviewee I8VW experienced an unpleasant situation in consultation, when the midwife made an unfortunate comment, thus demonstrating a lack of sensitivity and information about FGM and its approach: “The midwife found it very rare that performing a cervical smear would hurt so much and she told me: ’if the penis has fit there, it shouldn’t hurt so much’. I explained to her why it was so painful for me. Ashamed she apologised” (I8VW)

#### 3.2.2. Lack of Detection and Information

Participants reported that healthcare providers failed to detect FGM and its consequences. Practically all the women interviewed in Valencia reported that no professional asked them if they had undergone FGM at any time during their pregnancy or any other health visit: “No, no... they didn’t tell me anything about it, nobody” (I11VW), “No, no one has never asked me this” (I12VW). Some participants suggested that although some professionals may be knowledgeable about the practice, they do not ask because of embarrassment and a lack of skills to handle the situation: “I think they do know but they don’t ask, they may be ashamed…” (I17VW). This fact has an impact on the information provided and, consequently, the quality of care for these women, girls and families. On the contrary, all women who gave birth in a hospital in the British Health Care System were asked during pregnancy about FGM: “But when I was doing my booking, they asked me if I had FGM done” (I1LW), “During my first pregnancy they kept asking me if I had been circumcised” (I4LW).

#### 3.2.3. Stigmatizing and Over-Inquisitive Attitudes

Another important aspect to highlight is the stigma. To avoid stigmatization of different cultures and groups, professionals should demonstrate respect for different cultures and their ritual practices, which is wholly compatible with showing a frontal rejection of FGM. Professionals should avoid issuing blaming judgments to these women, since, as I1LW interviewee described, “she did not choose to be mutilated”. Statements from healthcare providers such as, “we have to refer you to social services” during the first pregnancy consultation were construed as very offensive, even more so for this specific interviewee, who as a midwife knew the action plan perfectly, but did not agree with the way in which the professionals had decided to act and communicate. This participant stated:

“But when I was doing my booking, they asked me if I had FGM done. I said yes, and they said that if I had a girl, they would have to refer me to social services… for child protecting issues. This was quite offensive because I don’t want anyone else what I have been through” (…). “It wasn’t my choice; it wasn’t me going to the doctor and saying: ‘I want FGM to be done on me’ (…). Instead of judging women, professionals should raise awareness, provide education and emotional support if women’s been traumatised”

(I1LW)

#### 3.2.4. Offering Reversal or Deinfibulation

Four of the participants were offered a reversal of FGM intrapartum which was accepted by two of them. These women who were offered a reversal attended the British healthcare service.

“I was in labour and the doctor came and told me that he will open before the baby came. I refused because I didn’t want it. But at the end I had caesarean not because pf the FGM but because the baby was stuck somewhere. With my second baby I also had caesarean”

(I2LW)

“They said to me: ‘You can’t deliver your baby unless we open you’. And I chose to be opened the day of delivery. I didn’t want to have a reversal in pregnancy, I wanted everything at the time of delivery, all together”

(I4LW)

None of the Spanish participants were offered a reversal during pregnancy. One Spanish participant requested surgical reconstruction, but she found that professionals did not always know the procedure to follow. Information on the possibility of undergoing reconstructive surgery was obtained in this case by friends, women in the same situation and the media (internet): “I requested it. I heard of it from a friend (…) I looked it up in ‘YouTube’, I looked for information so I found out what could be done (…) After asking different professionals, I found a midwife who referred me to the specialist doctor” (I14VW)

### 3.3. Tackling FGM

Under this main theme, the subthemes “education and awareness”, “speaking up”, “improving prevention” and “penalization” were derived.

#### 3.3.1. Education and Awareness

Participants emphasised the education and awareness of women and men -as a fundamental component in the practice maintenance-, both in current countries of residence and in countries of origin with prevalence of FGM.

“Oh God! Education, education, education. I can’t say it enough! And also educating men here but also back home”

(I1LW)

“To avoid this practice, women must go to school (…) When a woman is not educated, she cannot think, she cannot defend herself (…)”

(I9VW)

#### 3.3.2. Speaking Up

Furthermore, many of the participants emphasised the idea of speaking up about the practice and showing support for its eradication. Two of the interviewees described that after several years in Spain, they returned to their places of origin to publicise the consequences of FGM and work with the community towards its eradication, trying to generate action for social change.

“I explained to my mother the inconveniences and I have succeeded to prevent my daughter and nieces from FGM. It is possible! If there is a will, there is a way. I didn’t know that I could convince my mother either”

(I14VW)

“When I went back to my country, I gave a talk about FGM (…) at first, I looked as if I was no longer African, my mind had changed a lot”

(I14VW)

“I have also held a meeting with all the women in my town (…) Now I can say that 80% of the women in my town have abandoned the practice”

(I15VM)

#### 3.3.3. Improving Prevention

Another aspect that emerged during the interviews was the prevention of FGM in girls. Several quotes demonstrate that girls, despite residing in Europe, continue to be at risk of FGM when they travel to their country of origin: “One day I was talking to my mother, and she told me that she was waiting for my daughter to cut her clitoris with the others…” (I14VW).

#### 3.3.4. Penalization

Some interviewees proposed dealing with FGM by penalizing and prosecuting the practice. However, they also state that this is not always effective because although the performance of FGM may be punishable by law, the law is not always fulfilled: “Yes, yes, in my country it is prohibited. They do it, but it is prohibited. But it may be that one day it will end” (I11VW).

## 4. Discussion

In the present study, the experiences of women and men from FGM-affected countries were investigated via qualitative analysis and assigned to three main themes. As discussed in the interviews, the consequences of FGM are complex and affect different spheres of women’s and men’s lives along with their families. With regards to the consequences for women’s health which are described in our study, participants highlight both obstetric and gynaecological consequences and complications such as postpartum haemorrhage, perineal tears, completion of caesarean delivery, infertility, risk of infection and dysmenorrhea; genitourinary complications such as urinary tract infections and dysuria; consequences for sexuality, mainly dyspareunia, decreased or absent erotic desire, decreased quality of sexual intercourse and anorgasmia; and psychological consequences such as loss of self-esteem, feelings of humiliation, fear of social rejection and family disgrace, which coincide with several synthesis studies [13,35,36,37]. Death, as a major complication and consequence of FGM, was also referred to by several participants.

Although the consequences for women’s health appear to be the most predominant, socioeconomic consequences and those affecting men were also described by interviewees. Direct economic consequences on women and their families can originate fundamentally from the development of infections secondary to FGM that require expensive treatment, or the development of other complications that can lead to disability. In the long run and in certain contexts, this situation could lead to direct economic dependence on the husband or father [38]. In addition, the practice of FGM and the associated ceremony, can cause high costs that lead to family debt.

In many communities, FGM is a cultural requirement for girls who go into adulthood to acquire a certain social position and belong to a group. FGM is an important brand of social identity and not conforming to this can lead to social consequences, such as bullying, ridicule, social stigma, exclusion from the adult community, community events and social support, discrimination by peers, social rejection, loss of social status, increased isolation due to lack of marital capacity and family shame, as well as exclusion of the whole family from the social acceptance and welfare system of the community, as demonstrated by participants [38,39].

Regarding the consequences for men, those most mentioned included the pursuit of pleasure outside of the relationship, the fear of causing pain with penetration, or unsatisfactory sexual intercourse, as described previously [21,40,41].

Notwithstanding the health and social consequences acknowledged by most participants, we also found reports of women who did not associate any complications with the practice. In this way, Reig-Alcaraz [22] demonstrate a lack of self-awareness about the health implications for women who have undergone FGM. Our findings agree with previous research where Somali women expressed their feelings as: “It is normal” or “I am normal” [42,43]. For these women, there is no other way of being a woman and no other way of experiencing sexual intercourse and, motherhood, etc. They do not perceive the need to seek support, help or assistance, since FGM is commonly deemed intrinsic to a woman’s nature. Furthermore, a lack of education and misinformation about their own health accentuates this situation, which explains why women with a higher level of education advocate more strongly for the abandonment of the practice [44].

Regarding the healthcare received, the experiences of the respondents are varied. However, we observe clear differences between those residents in London and those interviewed in Valencia. For many of these women, their first contact with the health services in their host country is when they are pregnant [45]. During pregnancy, none of the women interviewed in Valencia reported that they were asked about FGM, even though having undergone FGM makes it more likely to experience obstetric complications [36] and special care is needed because the long-term health problems of FGM are in many cases irreversible. The impact of FGM during the birthing process should be sensitively discussed and a plan of care should be agreed to reduce fears about how the births will be managed [46]. All women, regardless of their country of origin, should be asked in their first pregnancy visits whether they have undergone FGM and this information must be recorded [36]. The detection of FGM was a causal finding during gynaecological examinations or during the birthing process in all cases attending the Spanish healthcare system.

In Spain, the study participants perceived a profound lack of knowledge about FGM by healthcare providers as evidenced in regional studies [24,47] that coincide with other European research [17,18]. In Valencia, less than a quarter of primary healthcare professionals correctly identified the typology of FGM, five percent correctly reported the countries where the practice is prevalent and only a third of the professionals were able to detect cases at risk of FGM [24]. Thus, most health providers do not know enough about FGM and are therefore uncertain of how to adequately deal with it. Moreover, participants reported a lack of information received during pregnancy and childbirth, as documented in other western countries [48]. In addition, interviewees reported negative experiences during vaginal examinations because professionals presented facial and verbal expressions denoting significant lack of knowledge in relation to the modification of the external genitalia. This fact has also been described in prior research [20,47,49].

Similarly, when requesting information on reconstructive surgery in Spain, there were severe difficulties in locating the appropriate information and referrals, as described in previous research [20]. This fact highlights the need for easily accessible educational resources and evidence-based guidelines to enable health professionals to reduce structural inequities and optimise health for women and girls who have undergone FGM.

On the other hand, women residing in the United Kingdom emphasised the high level of awareness and knowledge of healthcare providers. Based on the interviews with the participants, the professionals who attended them during their antepartum visits, labour and postpartum period, were fully aware of the established protocols and guidelines for action. Moreover, all women who gave birth in a hospital in the British Health Care System were asked during pregnancy about FGM. Despite this, women still felt discriminated against at times due to the stereotyping of healthcare providers and their insensitivity toward FGM. Interviewers also described a lack of understanding of cultural differences, perceiving the concerns of health professionals in relation to FGM as disproportionate. For example, some women perceived shaming and even threatening attitude in relation to the continuity of the practice of FGM for future newborns, without even having explored maternal intention in relation to the continuity or abandonment of the practice. In this sense, the experiences of migrant women collected by qualitative studies in countries with a large migrant population from countries affected by FGM reinforce a worrying lack of empathic care and sensitivity [45,46].

Finally, throughout the participants’ discourse, different strategies and actions to improve healthcare for women and girls affected by FGM as well as the prevention and eradication of the practice of FGM emerged. Participants highlighted the importance of educating and raising awareness among women and men both in their hosts countries and countries of origin [50,51,52,53]. Participants also mentioned the impact of speaking up and making FGM known globally. There are increasingly more initiatives to empower girls and parents to reject this harmful practice, pushing for deeper transformation in the community. However, empowerment and education come together.

Our findings also support a previously demonstrated need to improve prevention and detection strategies [21,47,50,54]. To build a relationship of trust with communities affected by FGM, healthcare providers must have an accurate understanding of the cultural background surrounding this practice, a working knowledge of the different types of FGM procedures that may be encountered and an awareness of both the acute and long-term complications. Asking routinely about FGM may encourage open communication and facilitate more positive experiences [43].

### Limitations

This study has certain limitations that require to be acknowledged. Firstly, one of the major limitations was searching for the study participants. When using the “snowball” technique, one of the possible biases involves the oversampling of a network of peers [27]. In addition, individuals who share economic or social activities and who present similar characteristics may end up having a greater representation in the sample [29]. To minimise this bias, multiple snowballs starting from different key informants were used, attempting to expand the scope of the research beyond a single network. Another limitation of this sampling method is that participants may hesitate to provide names of other people who have undergone FGM and on occasions, asking for it may have raised ethical problems for the participants. For this reason, key informants initially contacted potential participants. Those who agreed to receive the study information and to be contacted by the researchers were those who were ultimately approached for their participation in the study.

Secondly, because the nature of the subject can be very sensitive, it is possible that some of the interviewees have not been able to express their feelings and experiences with total spontaneity and freedom. Finally, it was also considered that participants may not have been completely truthful in some of the aspects discussed, because, among other reasons, FGM is a harmful practice punished both in the United Kingdom and in Spain.

## 5. Conclusions

The current study identifies health and social issues in migrant women and men affected by the practice of FGM from their own perspective. Migrant women residing in Spain perceived a profound lack of knowledge about FGM from healthcare providers and consequently stressed that the information received was insufficient. The findings also illustrate that sometimes participants encountered negative attitudes when accessing healthcare services in their host countries and for certain participants the language used by health care providers was seen as frightening or humiliating. Some women’s experiences suggest a concerning absence of sensitive and empathetic care and a more woman-centred and human rights-based approach is recommended.

Our findings disclose the need to improve training and institutional plans to address structural and attitudinal barriers to health equity across migrant families in their host countries. This study may contribute to making visible this unknown practice among health providers and may serve as a basis to formulate strategies aimed at strengthening the care of women and girls affected by FGM from a comprehensive, respectful, cultural and gender perspective, while also being effective in eliminating the physical and psychological consequences of FGM and reducing health inequalities for migrant women and girls.

Trying to deal with the crisis of violence against women this study provides insights from the perspective of women who have been affected directly by the issue, through the discussion of personal experiences related to the provision of care. It also offers a broad and holistic understanding about the phenomenon studied, which can inform professionals about the realities of the practice of FGM. Such insights are vital to provide women-centred care, particularly for women and girls from vulnerable groups whose voices are often unheard.

## Figures and Tables

**Table 1:** Types of female genital mutilation [2].

Type	Description
I	Clitoridectomy
	This is the partial or total removal of the clitoris and in very rare cases, only the prepuce.
II	Excision
	This is the partial or total removal of the clitoris and the labia minora with or without excision of the labia majora.
III	Infibulation
	This is the narrowing of the vaginal opening through the creation of a covering seal. The seal is formed by cutting and repositioning the labia minora or labia majora, sometimes through stitching with or without removal of the clitoris.
IV	Others
	This includes all other harmful procedures to the female genitalia for non-medical purposes, e.g., pricking, piercing, incising, scraping and cauterizing the genital area.

**Table 2:** Sociodemographic and FGM characteristics of participants (individual interviews).

Number	Sex	Age	Place of Origin	Profession	Ethnicity	Residence	Years Out of Country of Origin	FGM	Type of FGM	Age of FGM (Years)
I1LW	Woman	28	Kismaayo, Somalia	Midwife	-	London	22	Yes (in Italy)	I	6
I2LW	Woman	43	Kismaayo, Somalia	Health Care Assitant	-	London,	17	Yes	II-III (Pharaon)	6–7
I3LW	Woman	44	Mogadishu, Somalia	Health clinic assistant	-	London	5	Yes	II-III (Pharaon)	-
I4LW	Woman	28	Mogadishu, Somalia	Housewife	-	London,	12	Yes	III (oni)	9
I5LW	Woman	36	Mogadishu, Somalia	Housewife	-	London,	13	Yes	II	5–6
I6VW	Woman	43	Senegal	Catering	-	Valencia	9	No	-	-
I7VM	Man	47	Cameroon	Catering	Bamileke	Valencia	16	-	-	-
I8VW	Woman	35	Dakar, Senegal	Hospitality	Wolof	Valencia	11	No	-	-
	* sister in law		*Dakar, Senegal (originally from Guinea)*	*-*	*-*	*France*	*-*	*Yes*	*Uncertain*	*12*
I9VW	Woman	39	Nigeria	Hospitality	Ibu	Valencia	11	No	-	-
I10VW	Woman	40	Bata, Ecuatorial Guinea	Hospitality	Fang	Valencia	18	No	-	-
	* female relative		*Nigeria*	*-*	*-*		*-*	*Yes*	*Uncertain*	*-*
I11VW	Woman	43	Uagadugú, Burkina Faso	Cleaning staff	Mossi	Valencia	10	Yes	I	1
I12VW	Woman	34	Mali	Housewife	Bambara	Valencia	8	Yes	Uncertain	3–4
I13VW	Woman	31	Edo, Nigeria	Housewife	-	Valencia	5	Yes	Uncertain	<1
I14VW	Woman	29	Kayes, Mali	Technical staff in Foundation	Mandike	Valencia	8	Yes	III	1 week
I15VM	Man	53	Malí	Seasonal worker	Bambara	Valencia	7	*-*		*-*
	* his wife	44	Malí	-	Bambara	Valencia	3	*Yes*	*Type II*	*-*
I16VW	Woman	28	Bamaku, Mali	Student	Fulani (Peul)	Valencia	8	Yes	Uncertain	<7
I17VW	Woman	25	Kayes, Mali	Housewife		Valencia	2	Yes	Uncertain	1 month
I18VW	Woman	26	Rural area, Mali	Housewife		Valencia	1	Yes	Uncertain	1 month

* Wife and female relatives who had FGM performed. I8VW, I10VW and I15VM included because had been in close contact with the phenomenon studied.I: interview: V: Valencia; L: London; W: woman; M: man.

**Table 3:** Profile of participants included in the focus group.

Interview	Age	Country of Origin	Years out of Country of Origin	Marital Status	FGM, Type
IG1VW	19	Nigeria	2	Single	Yes, uncertain
IG2VW	19	Nigeria	5	Single	Yes, uncertain
IG3VW	31	Nigeria	4	Single	Yes, uncertain
IG4VW	22	Nigeria	1	Single	Yes, uncertain
IG5VW	24	Nigeria	3	Single	Yes, uncertain

I: interview; G: Grupal; V: Valencia; W: woman.

**Table 4:** Themes and subthemes emerging from the data.

Theme	Subtheme
Consequences of FGM	Obstetric consequences Genitoruinary complications Pain Sexual complications Psychological and social consequences Lack of insight Men’s consequences
Healthcare received	Unacquainted professionals Lack of detection and information Stigmatizing and over-inquisitive attitudes Offering reversal or deinfibulation
Tackling FGM	Education and awareness Speaking up Improving prevention Penalization

**Table 5:** Obstetric and gynaecological consequences of FGM.

Obstetric-Gynaecological Complications	
Postpartum haemorrhage	“I started working in a maternity ward (…) when a pregnant woman arrived for birth it was a disaster (…) two women in front of me lost a lot of blood…” (I15VM) “Two girls from my town, 18 and 22 years old died in childbirth (…) in my town there isn’t a blood bank, there is nothing at all, if your wife has a problem with childbirth you have to take the woman by bicycle to the nearest maternity hospital, which is 8, 10, 20 km away from town… “ (I15VM)
Perineal tears	“It was very painful, that’s what I always say. I asked the midwife then how many stitches I had, but they could nou be counted. They gave me a lot of stitches inside and outside” (I14VW)
Infection	“There are many complications for women (…) first, there are many hygienic infections” (I8VW) “Strong pain ... but also if you are unlucky you get an infection...” (I6VW)
Arm palsy	“The problem was with my third baby (…), to get him out they forced his arm… the midwife squeezed a lot and his arm ended up broken. He has arm paralysis” (I11VW)
Preterm birth	“They were very preterm; they were born at 23 weeks” (I16VW)
Infertility	“Yes, yes, we were trying for a while, with the second insemination I got pregnant” (I16VW)
Death	“When you are pregnant… labour is usually very difficult, there are even girls who die giving birth” (I8WM) “At times there are girls who bleed a lot and sometimes they die” (I11VW)

**Table 6:** Quotations related to pain associated to the practice of FGM.

Pain	
Dysmenorrhea	“I was lucky because I never had problems with my periods, the man who was doing it was a doctor, so he didn’t cut us like other people from outside the city” (I2LW)
Dysuria	“The first time I went for a wee it was very painful. I didn’t want to go to toilet, I was holding my wee… I was crying I will never forget that moment” (I2LW) “Bad things, pain for wee at the beginning. After 2 weeks, it was normal” (I5LW)
Dyspareunia	“Period problems, having sex is very painful…” (I2LW) “She has 3 children but with her husband during intercourse she is always screaming” (I8VW)
Pain during birth	“I didn’t feel it, but they say that when you are giving birth there are women who find it much more difficult (…) it hurts more when giving birth (…) They say that there are births that last longer and are harder” (I11VW) “I have explained to him the inconveniences that women have when giving birth because they see it the opposite of the ones here; they think that women who are not mutilated at the time of giving birth they will suffer a lot, but it is totally the opposite” (I14VW)
